# Fatal Hemothorax Caused by Pseudomesotheliomatous Carcinoma of the Lung

**DOI:** 10.4061/2011/836054

**Published:** 2011-06-27

**Authors:** Petur Snaebjornsson, Cornelis G. Vos, Koen J. Hartemink, Rutger J. Lely, Setareh M. Samii, Katrien Grünberg, Marinus A. Paul

**Affiliations:** ^1^Department of Pathology, VU University Medical Center, De Boelelaan 1117, 1081 HV Amsterdam, The Netherlands; ^2^Department of Surgery, VU University Medical Center, 1081 HV Amsterdam, The Netherlands; ^3^Department of Radiology, VU University Medical Center, 1081 HV Amsterdam, The Netherlands; ^4^Department of Pulmonology, VU University Medical Center, 1081 HV Amsterdam, The Netherlands

## Abstract

We present a case of a poorly differentiated pseudomesotheliomatous carcinoma originating in the lung, which was manifested with the distinctly rare complication of massive true hemothorax and persistent blood loss that proved rapidly fatal in spite of surgery. Pseudomesotheliomatous carcinoma of the lung and neoplasia-associated hemothorax are reviewed and discussed.

## 1. Introduction

Massive, spontaneous hemothorax, is an unusual and rare presenting sign of lung cancer [1]. This complication has, to the best of our knowledge, not previously been reported concomitant with pseudomesotheliomatous carcinoma of the lung. The term pseudomesothelioma refers to tumors that simulate malignant mesothelioma clinically, radiologically, or pathologically [2]. 

We hereby present a case of a poorly differentiated lung carcinoma with extensive dissemination along the pleural surfaces and interstitial growth pattern, including angioinvasive growth, which manifested itself as massive spontaneous true hemothorax. Immediate and repeated thoracic surgery could not prevent death. The literature on specific characteristics of lung cancer as a cause of hemothorax is reviewed and discussed.

## 2. Clinical History

**Figure 1:**
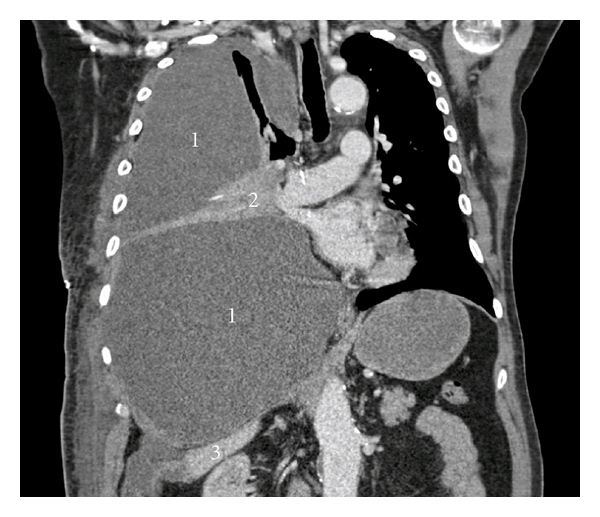
Coronal CT scan image demonstrating the hemothorax (1) with atelectasis of the right lung (2), mediastinal shift to the left, and downward displacement of the liver (3), but no visible intrapulmonary lesions or other signs of malignancy.

A 74-year-old Caucasian male was presented to the Emergency Department with progressive dyspnea, existing for 5 days. There were no symptoms of cough, hemoptysis, or fever and no preceding trauma. His medical history was significant for a myocardial infarction and the placement of a pacemaker because of bradycardia. In addition, he had a ruptured aneurysm of the abdominal aorta 11 years ago, for which he was treated with aortic bifurcation prosthesis. He used acetylsalicylic acid but no other anticoagulant medication. He was a smoker (50 pack years), and there was no known exposure to asbestos. Clinical examination revealed tachypnea with a respiratory rate of 26 breaths per minute. Vital signs were all normal, and oxygen saturation was 100% with 4 liters of oxygen per minute. Breath sounds were absent on the right hemithorax. A chest radiograph demonstrated a large amount of fluid in the right pleural cavity. Laboratory analysis showed anemia (hemoglobin [Hb] of 5.6 mmol/L, normal range 8.5–11 mmol/L). White cell count, C-reactive protein, and coagulation time were normal. A chest tube was placed, and 1200 mL of blood (Hb 3,6 mmol/L) was drained immediately. In the first 24 hours, a total of 3 liters of blood was drained. A chest computer tomography (CT) scan confirmed the hemothorax (Figure 1), but no site of active bleeding or tumor could be identified. There were no pathological mediastinal lymph nodes.

Blood loss persisted, demonstrated by a fall in Hb, despite administration of 5 packed red blood cells. In addition, the patient developed a fever (38.6°C), and his C-reactive protein and white cell count increased, suggesting an infected pleural hematoma. Therefore, a right posterolateral thoracotomy was performed and a large hematoma was removed. Bacterial cultures remained negative. A bleeding ulcer was found in the right upper lobe and was removed by wedge resection. No other pleural lesions were observed at careful inspection.

Initially, the patient recovered uneventfully. On the 10th postoperative day, he acutely developed a hypovolemic shock, again due to a hemothorax. A CT scan with intravenous contrast showed a hemothorax but no focus of active bleeding. The patient was stabilized with fluid administration and packed red blood cells and underwent a rethoracotomy. This time the pleura was thickened with an irregular surface, macroscopically resembling carcinomatosis. The pleural surface showed diffuse bleeding, and 1.7 L of blood was evacuated from the pleural cavity. To minimize tumor-load and to obtain pleurodesis, a total pleurectomy was performed, although there was no proof of malignancy at that time. Intraoperative frozen section analysis was not performed during this second operation since it was not believed to change the course of treatment at that time.

On the third postoperative day, the patient developed respiratory distress due to right lower lobe pneumonia. The patient deteriorated despite antibiotics and supportive treatment. Because of his locally advanced malignancy and poor condition, both the patient and family requested to discontinue treatment. The patient died 21 days after initial presentation. A postmortem examination was not performed. 

## 3. Materials and Methods

The specimens were fixed in 4% buffered formalin, routinely processed, and paraffin embedded. 4 *μ*m-thick sections were stained with hematoxylin-eosin (HE) and the following standard stains: PAS, PAS-diastase, and elastica van Gieson. Automated immunohistochemistry was performed on a Ventana Nexus en Bond Max automated immunostainer using antibodies for CAM5.2 (B&D), AE1/3 (Novocastra), MOC31 (Novocastra), CK7 (Novocastra), CK20, 34*β*E12, CK14 (Neomarkers), CK19, EMA, CEA (BioGenex), TTF-1, SP-A, CD56 (Novocastra), chromogranin, synaptophysin, calretinin, thrombomodulin, D2-40, CD31, CD34, Factor VIII, E-cadherin, Mib-1, thyroglobulin, P53, S100, vimentin (VUmc), and CDD99 (all from Dako if not otherwise indicated) and was applied according to standard protocols. Appropriate positive and negative tissue control samples were used.

## 4. Results

### 4.1. Macroscopic Findings

The initial wedge resection showed a slightly greyish thickening of the visceral and parietal pleura at gross inspection. An ulceration of the visceral pleura with adherent blood was recognized. Underneath the visceral pleura an irregular and poorly defined greyish consolidation of approximately 2 cm was seen. There were no other detectable masses present. There was no connection to large airways.

### 4.2. Histological Findings

**Figure 2:**
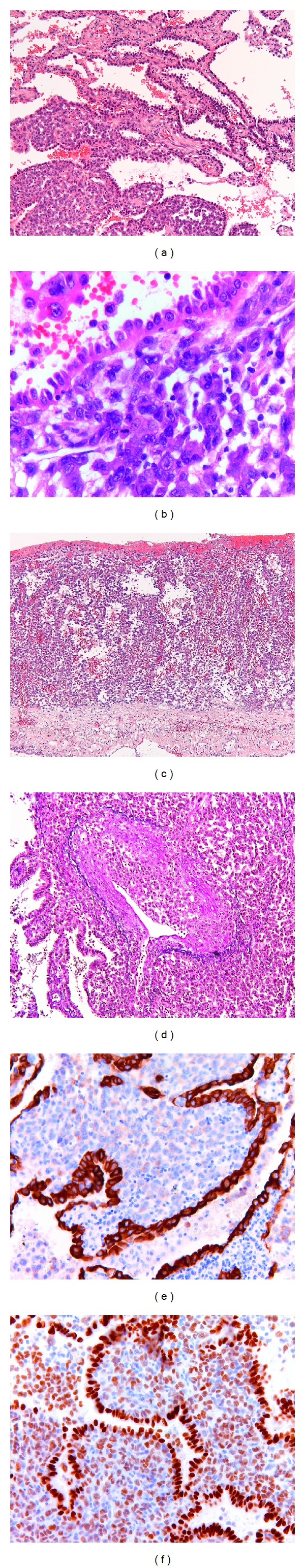
Histological and immunophenotypic features of the tumor: interstitial infiltrating growth pattern of a poorly differentiated carcinoma ((a), detail in (b)) with atypical BAC-like pneumocytes lining the alveoli in (b) and around (a) the interstitial component of the tumor (HE). The visceral pleura shows a cake of highly vascularized and poorly cohesive carcinoma ((c), HE), with cytonuclear features similar to the interstitial infiltrating tumor in the lung. (d) The tumor invades into a medium-size vessel (elastica van Gieson stain). There is strong immunoreactivity for CAM5.2 (e) and TTF-1 (f) in the atypical BAC-like component and variable immunoreactivity for CAM5.2 (e) and TTF-1 (f) in the infiltrating carcinoma.

Microscopy revealed features of a diffusely growing discohesive carcinoma, exclusively growing in the alveolar interstitium, thus expanding it, while leaving the original alveolar architecture intact (Figure 2). There was local ulceration of the pleura, while, beyond this ulcer, the tumor formed a thick cake of discohesive tumor cells lining the pleural membrane (Figure 2(c)), with only focal, microscopic invasion into the fatty tissue of the parietal pleura.

The tumor consisted of atypical, moderately polymorphous, and irregularly shaped tumor cells with marked discohesiveness. They featured scant eosinophilic cytoplasm and irregularly contoured and hyperchromatic nuclei, often containing one or more prominent nucleoli (Figure 2(b)). There were many mitoses and apoptoses present, but necrosis was not observed. No squamous or glandular differentiation was observed, and mucin stains (PAS-D and alcian blue) were negative. Within the tumor, there were multiple small blood-filled clefts and blood lakes. Angioinvasion in medium-sized vessels, including an artery, was demonstrated (Figure 2(d)).

Of note, the broadened alveolar septa were lined by markedly atypical epithelial cells, yet less atypical than the interstitial carcinoma (Figures 2(a) and 2(b)). The atypia of the lining cells extended beyond the tumor front, showing a sharp demarcation with normal type I pneumocytes (Figure 2(a)), a feature characteristic to nonmucinous adenocarcinoma in situ with lepidic growth pattern (former bronchioloalveolar carcinoma, BAC) [3].

### 4.3. Immunohistochemical Findings

There was partial weak positivity for the keratin proteins CAM5.2 (Figure 2(e)), CK14, and CK19, and strong positivity for vimentin, CD99, and AE1/3. The tumor cells were nonreactive with EMA, CEA, MOC31, CK7, CK20, 34BE12, SP-A, and S100 and only mildly reactive with P53 and CD56. The neuroendocrine markers chromogranin and synaptophysin were negative. Calretinin, thrombomodulin, and D2-40, frequently positive in malignant epitheloid mesothelioma, were negative. Because of its high vascularity, angiosarcoma was considered in the differential diagnosis, but markers for vascular differentiation (CD31, CD34, factor VIII) were negative in the tumor cells. These markers did reveal the presence of a fine network of thin-walled vessels within the tumor, including many sinusoid-like vascular spaces. The tumor showed near-diffuse staining for TTF-1, varying from negative to strongly positive (Figure 2(f)). TTF-1 positive staining has to our knowledge not been described in angiosarcomas or mesotheliomas. On the basis of these findings, a histological diagnosis of poorly differentiated adenocarcinoma of the lung was made. Consistent with discohesive growth the tumor cells were negative for E-cadherin. Mib-1 was positive in most tumor cells, confirming high proliferative activity. Thyroglobulin was negative.

The atypical alveolar lining cells were uniformly and strongly positive for TTF-1, CAM5.2, CK7, CEA, EMA, beta-catenin, and surfactant A (Figures 2(e)–2(f)). There was a diffuse weak positivity for p53.

### 4.4. Molecular Findings

No mutation in EGFR exons 19–21 and KRAS exons 1-2 could be detected (PCR and sequencing).

The second pleural resection showed an identical picture of pleural cake with only focal microscopic invasion. As no other lesions of the pleura were observed at surgery 10 days before, this finding supports the suspected rapid progression of the tumor.

## 5. Discussion

Many tumors feature metastases to the pleural membranes in the late stage of the disease, but predominant serosal involvement mimicking malignant mesothelioma is a rare presenting sign [2]. Various malignant pseudomesotheliomatous tumors have been reported, including different types of primary lung carcinomas [2], a variety of sarcomas [4], thymic epithelial tumors [4], melanoma [5], hematopoietic neoplasms [4, 6], and many metastatic tumors [2, 4]. Pseudomesotheliomatous carcinomas originating in the lung tend to be peripherally located and are characterized by extensive pleurotropic growth and inconspicuous parenchymal involvement [7]. Pseudomesotheliomatous lung carcinomas are a heterogenous group of tumors. Attanoos and Gibbs reported 47 cases of primary pulmonary carcinoma with extensive pleural spread of which 70% were adenocarcinomas [2]. Other reported types of lung carcinomas mimicking malignant mesotheliomas include various high-grade carcinomas such as pleomorphic carcinoma, small-cell carcinoma, basaloid carcinoma, carcinosarcoma [2], high-grade neuroendocrine carcinoma [8], and large-cell carcinoma [9]. Also, squamous cell carcinoma, signet ring-cell carcinoma [10], and atypical carcinoid [11] have been reported. Pleural pseudomesotheliomatous tumors have poor prognosis with a median survival of 8 months, similar to stage IV non-small cell lung cancer [2, 12].

The presented case featured subpleural interstitial growth and a pleural spread of discohesive tumor cells. In addition, there were atypical BAC-like pneumocytes lining the alveoli in and around the interstitial component of the infiltrative tumor. These cells showed no nuclear stratification, (micro)papillary or glandular differentiation present but were strongly positive for TTF-1 and keratin markers, suggestive of a BAC-component in a poorly differentiated adenocarcinoma. Whether this represents a true in situ component of the same adenocarcinoma, or reactive atypia, cannot be distinguished with certainty. If considered to be in situ carcinoma, then it can only be speculated as to whether the interstitial component or the in situ component came first, or even whether there could be an ongoing interchange between the two. The constellation of an interstitial TTF-1 positive tumor together with atypia of the alveolar lining cells is rarely observed in lung carcinomas. This combination, however, is reminiscent of pneumocytoma [13]. Clearly, this case differs from pneumocytoma in that it represents a high-grade and rapidly progressive tumor, a feature that has not been described in pneumocytoma.

Our patient presented with a massive hemothorax which required thoracotomy and wedge resection to control the bleeding. After removal of the ulcerating lung lesion, the hemorrhage recurred, resulting in severe anemia together with circulatory failure and postoperative infection, causing death 21 days after presentation. Bleeding from a lung cancer usually presents as hemoptysis resulting from necrosis and vascular rupture or tumor erosion of pulmonary vessels. Approximately 7% of patients with lung cancer manifest with hemoptysis, and centrally located bronchial tumors are much more frequently associated with hemoptysis than peripheral tumors [14, 15]. Patients with pseudomesotheliomatous carcinoma have not been reported to present with hemoptysis [16].

Lung carcinoma is a distinctly uncommon cause of hemothorax [17], and we found only one case reported hitherto [1]. Spontaneous neoplasia-associated hemothorax has most commonly been associated with neurofibromatosis type 1, angiosarcoma, and hepatocellular carcinoma, the latter known for its vascular stroma [17]. Mediastinal tumors have also been reported to cause hemothorax [17]. Bleeding caused by these tumors has been attributed to several mechanisms. One is direct oozing or exsanguination of the tumor into the pleural space, and another is acute bleeding due to rupture of the primary tumor [18]. We believe that both mechanisms could explain the intractable bleeding in the present case, considering the serosal ulceration of the primary lesion and multiple small blood-filled clefts and vessels between the discohesive tumor cells. In addition, tumor invasion into larger vessels including arteries, causing vascular lesions, may have played a role [19].

Pleural effusions are often seen together with pseudomesotheliomatous carcinomas of the lung [16, 20], and these are frequently blood-stained (serosanguinous). True hemothorax is defined as pleural fluid with a hematocrit equal to or greater than 50% of the blood hematocrit [17]. Values of less than 50% have sometimes been called bloody pleurisy [18]. Measuring the hematocrit (or hemoglobin concentration) to distinguish between true hemothorax and bloody pleurisy is important as these two conditions have different etiologies and therapeutic options [17].

Primary treatment of true hemothorax is by chest tube drainage, which evacuates blood and prevents the formation of a clotted hemothorax with restricted lung function. This approach decreases the risk of empyema, and drainage allows monitoring the amount of blood loss. However, in case of persistent blood loss, as determined by chest drain production or hemodynamic instability, surgical exploration should be considered. Treatment of neoplasia-associated hemothoraces depends on the tumor and frequently involves resection of the lesion. The prognosis of malignancy-associated hemothorax, as with pseudomesotheliomatous carcinoma, is poor [17].

We have presented a case of a rare type of poorly differentiated pseudomesotheliomatous carcinoma originating in the lung, complicated by massive true hemothorax and persistent blood loss that proved rapidly fatal in spite of surgery. Awareness of this rare complication is important for an early diagnosis and intervention. Continued case reporting is necessary to determine which protocols would be optimal for treating this tumor.
